# The metal cofactor: stationary or mobile?

**DOI:** 10.1007/s00253-024-13206-2

**Published:** 2024-06-24

**Authors:** Peter-Leon Hagedoorn, Martin Pabst, Ulf Hanefeld

**Affiliations:** Afdeling Biotechnologie, Technische Universiteit Delft, Van der Maasweg 9, Delft, 2629 HZ The Netherlands

**Keywords:** Metal cofactor, Class II aldolase, Xylose isomerase, Medium-chain dehydrogenase, Metal movement

## Abstract

**Abstract:**

Metal cofactors are essential for catalysis and enable countless conversions in nature. Interestingly, the metal cofactor is not always static but mobile with movements of more than 4 Å. These movements of the metal can have different functions. In the case of the xylose isomerase and medium-chain dehydrogenases, it clearly serves a catalytic purpose. The metal cofactor moves during substrate activation and even during the catalytic turnover. On the other hand, in class II aldolases, the enzymes display resting states and active states depending on the movement of the catalytic metal cofactor. This movement is caused by substrate docking, causing the metal cofactor to take the position essential for catalysis. As these metal movements are found in structurally and mechanistically unrelated enzymes, it has to be expected that this metal movement is more common than currently perceived.

**Key points:**

• *Metal ions are essential cofactors that can move during catalysis.*

• *In class II aldolases, the metal cofactors can reside in a resting state and an active state.*

• *In MDR, the movement of the metal cofactor is essential for substrate docking.*

**Graphical abstract:**

**Figure:**
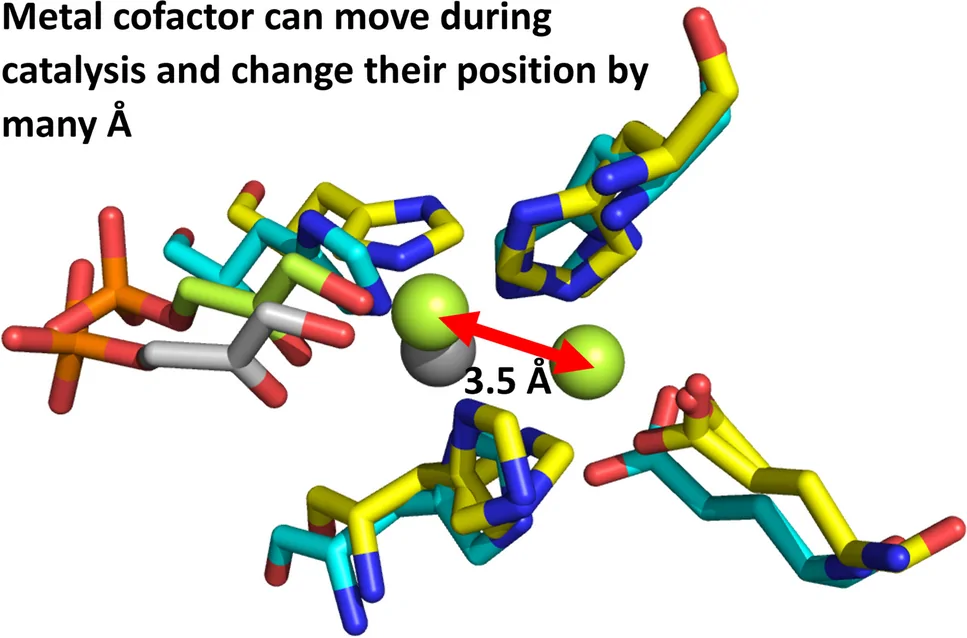

## Introduction

Metals are omnipresent in nature. All the soluble and bioavailable metals have been associated with proteins to induce both stability and catalytic activity (Kunkle and Skaar 2023; Waldron and Robinson 2009). About a third of all enzymes are metalloenzymes and in many of these metalloenzymes, the metal is the cofactor that brings catalytic activity. The metal can induce oxidation and reduction activity, both via single electron radical processes as well as electron pair transfer as for instance in the case of hydride shifts. Isomerization and bond formation, including Lewis acid catalysis, extend the scale of reactions catalyzed by metalloenzymes even further. The metal can be complexed directly by amino acid residues of the protein or by a prosthetic group such as porphyrin-derived ligand systems (Frey and Hegeman 2007). Throughout all these conversions, one observation can be made: the metal site is perceived as stationary throughout the catalytic cycle (Ben-David et al. 2013; Torrance et al. 2008), just like in homogeneous catalysis (Hanefeld and Lefferts 2018). The substrate enters the enzyme active site, is activated by the metal, and after conversion to the product, leaves the enzyme. Substrate and product are mobile while the metal is static during the catalytic cycle. This is in great contrast to the metal mobility in protein stabilization, where in particular Zn is known to greatly influence protein structure via alternating binding modes (Maret and Li 2009). A comprehensive analysis of metalloenzymes shows that similar metal mobility can also be found in some metalloenzymes. Here, we review examples were the assumption of a stationary metal site is incorrect and look at possible functional roles of the mobility of the metal cofactor. We here discuss them in chronological order of discovery.

## d-Xylose isomerase

**Scheme 1:**
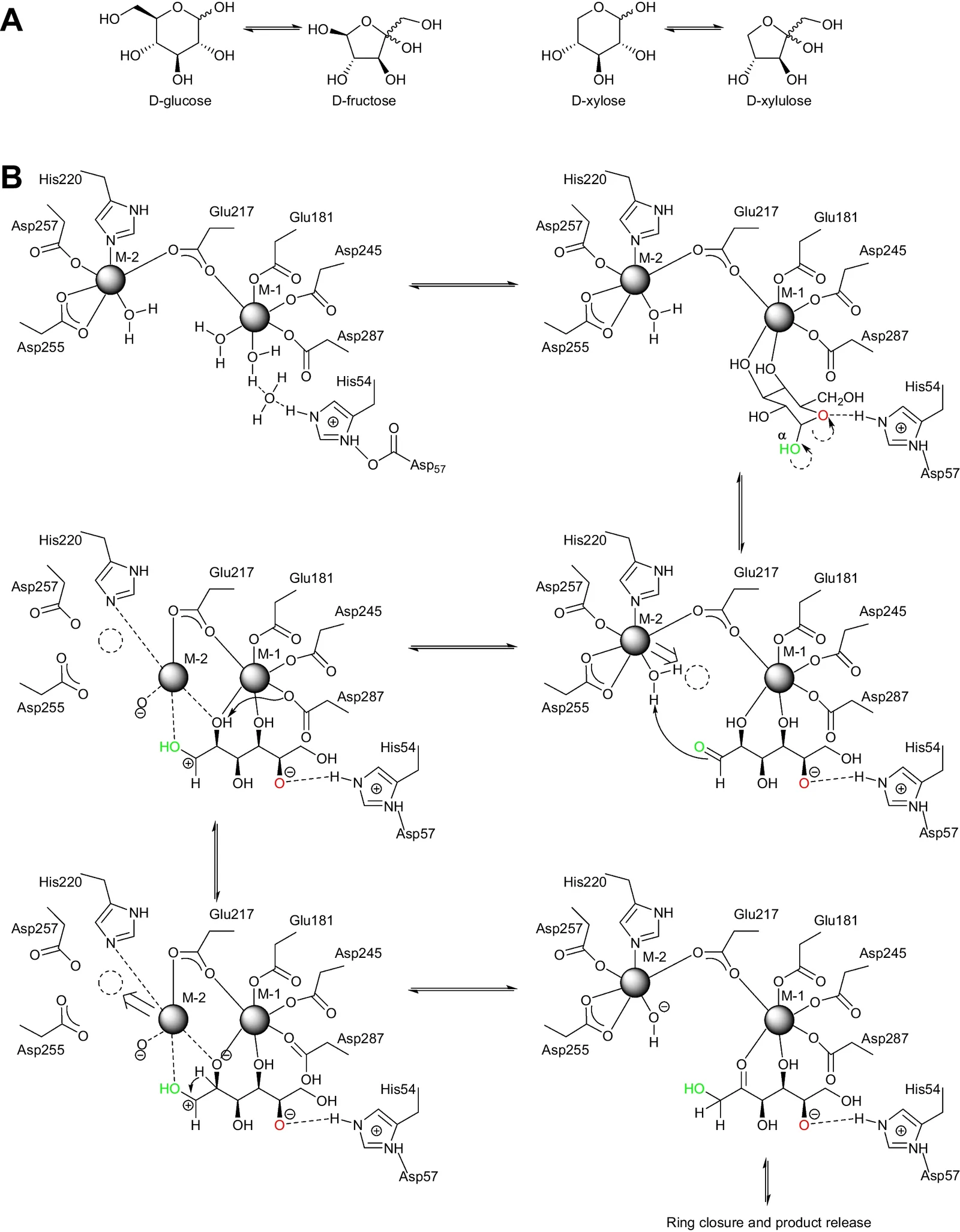
(**A**) XI catalyzes the isomerization of d-xylose into d-xylulose and d-glucose into d-fructose. The reaction proceeds via a ring-opening step followed by the actual isomerization and is concluded by the cyclization of the product. (**B**) Suggested reaction mechanism based on combined X-ray and neutron diffraction studies of XI from *Streptomyces rubiginosus* (*Sr*XI) (numbering of the active residues is different to *So*XI). In addition to the movement of M-2, it was proposed that a water molecule acts catalytically as proton donor and that the hydroxy group at C-5 bears a negative charge after ring opening of the sugar (Kovalevsky et al. 2010)

The d-xylose isomerase (XI), also known as glucose fructose isomerase, converts d-xylose into d-xylulose and d-glucose into d-fructose (Scheme 1) (Lavie et al. 1994). For the latter reaction, XI is applied industrially in the High-Fructose Corn Syrup (HFCS) production. With more than 10,000,000 tons of HFCS produced per year, this is the largest biocatalytic industrial process (DiCosimo et al. 2013). The enzyme’s intriguing mechanism of the two-stage reaction has attracted much interest. The ring opening of the substrate is followed by what in solution chemistry formally is a Lobry de Bruyn–Alberda van Ekenstein isomerization, then the product cyclizes again and leaves the active site. Intriguingly, the enzymatic mechanism follows a different pathway than the acid or base catalyzed Lobry de Bruyn–Alberda van Ekenstein isomerization, as will be discussed below.

**Fig. 1:**
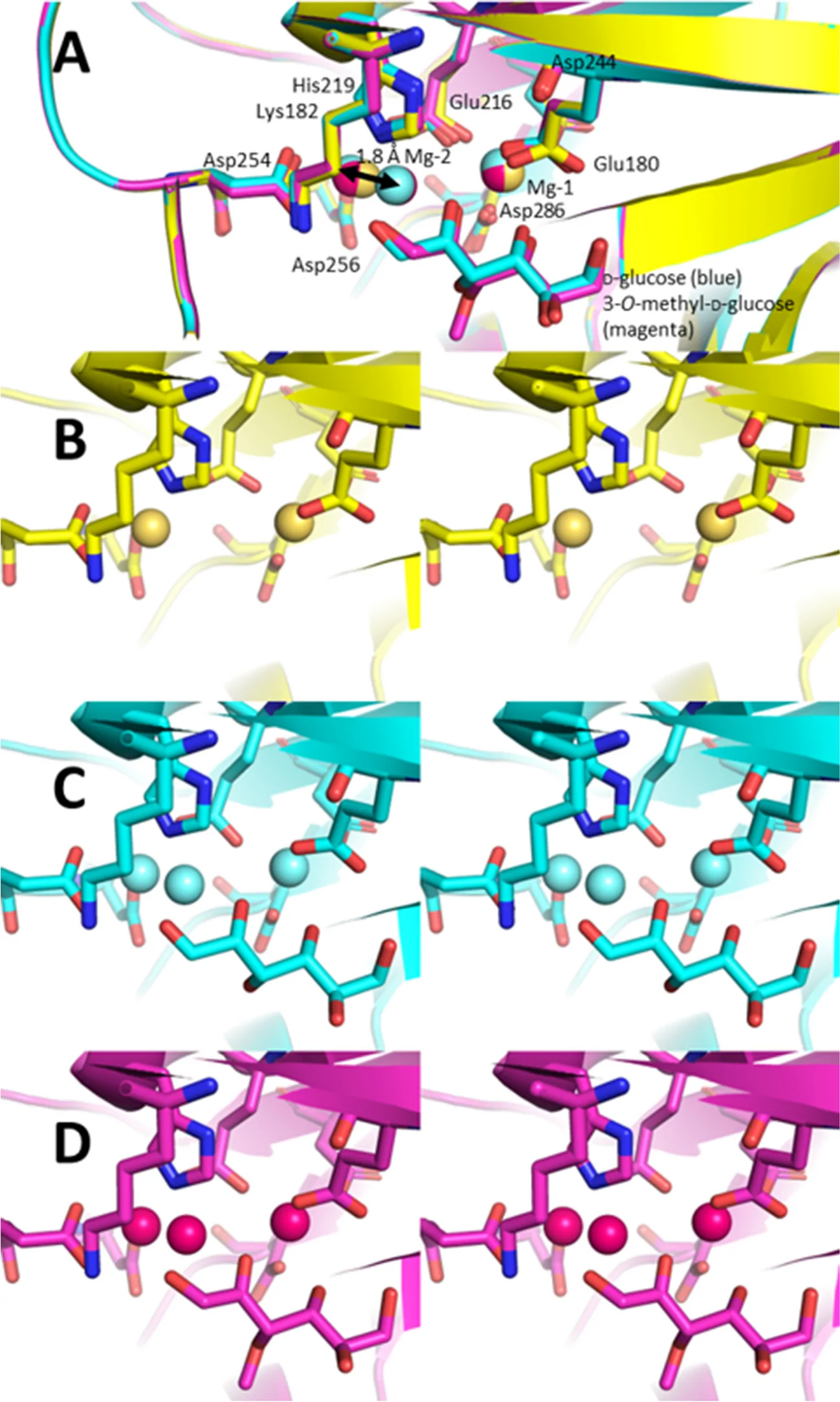
*So*XI was crystallized (PDB-ID 1XYA, yellow) and soaked with d-glucose (PDB-ID 1XYB, blue) and 3-*O*-methyl-d-glucose (PDB-ID 1XYC, magenta). (**A**) Overlay of all three structures shows the 1.8 Å movement of Mg-2 while all key residues do not shift significantly. (**B**) Wall-eyed stereoview of XI in resting state (PDB-ID 1XYA) with both Mg ions each in their resting position. (**C**) Wall-eyed stereoview of XI with d-glucose (PDB-ID 1XYB). The electron density does not allow to distinguish between glucose and fructose. A significant part of Mg-2 has shifted from its resting state to coordinate the oxygen of the aldehyde group of d-glucose. (**D**) Wall-eyed stereoview of XI with 3-*O*-methyl-d-glucose (PDB-ID 1XYC). Also here the shift of Mg-2 is clearly visible

To catalyze both ring opening and isomerization, the enzyme utilizes two divalent metal ions. In the key publication revealing the metal shift, the divalent metal is Mg(II) (Lavie et al. 1994). X-ray crystallographic studies of *Streptomyces olivochromogenes* XI (*So*XI) revealed that one of these Mg ions is stationary but the other moves 1.8 Å to stabilize the open chain form of the substrate and enable the isomerization. In the resting state, both Mg ions are clearly defined. Mg-1 is coordinated by four acid groups (Asp286, Asp244, Glu180, and Glu216); Glu216 acts also as ligand for Mg-2. Mg-2 additionally is held in position by two other acid residues (Asp254 and Asp 256) and His219 (Fig. 1). Lys182 acts as base in isomerization reaction.

By combining X-ray and neutron diffraction studies, a more detailed insight into the mechanism of the two-step catalysis could be obtained (Kovalevsky et al. 2008, 2010). In these studies, XI from *Streptomyces rubiginosus* (*Sr*XI) was used and again Mg is the metal at both metal binding sites. Based on seven crystal structures, replacing the active metals with inactive metals such as nickel and cadmium and additional D_2_O exchange, as well as locating hydrogen via neutron diffraction, allowed to identify the role of the different residues. Therefore, the metal is here indicated as M. M-1 binds to four acid groups (Glu181, Glu217, Asp245, and Asp287 as well as two water molecules); again one of them, here Glu217 is shared with M-2. M-2 is also coordinated by His220, Asp255 (bidentate), Asp257, and the catalytic water (Scheme 1B).

The two metal ions act in concert to enable the catalysis. The α-pyranose enters the active site and doubly protonated His54 (proton from Asp57) induces a shift of a hydrogen from the α-hydroxy group and the following cascade opens the ring. The aldehyde is thus released from the hemiacetal and the hydroxy group of C-5 is deprotonated in the open chain. The M-2 then shifts to activate the aldehyde and the catalytic water is deprotonated. This generates the positive charge on C-1 enabling the hydride shift, which occurs from C-2 after deprotonation of the C-2 hydroxy group by Asp287. Subsequently, the isomerized sugar can again cyclize and leave the active site (Kovalevsky et al. 2010). In an extension of this study, also other sugars and their isomerization were investigated (Langan et al. 2014).

Very recently, it was shown that the metal motility of M-2 in XI can reach even further. Serial crystallography enabled the study of xylitol-bound *Sr*XI (Nam 2021). When the inhibitor xylitol, i.e. the reduced form of the substrate, binds, the M-2 can even dissociate leading to deactivation of the enzyme. This is relevant in the HFCS production. Even low rates of deactivation have a significant influence on the industrial scale application of the enzyme (Nam 2022).

The ring opening and isomerization mechanism of XI are more widely spread in sugar metabolism. Also the l-rhamnose isomerase works according to this two-metal mechanism with a catalytic water molecule. In this case, the divalent metal is Mn. Here, the static M-1 always coordinates the C-2 O and the C-3 hydroxy group, both during catalysis of the ring opening and isomerization. M-2 coordinates the catalytic water, enables the isomerization, and displays a shift (Yoshida et al. 2010, 2013, 2024). Remarkably also for the unrelated enzyme bacterial tyrosinase which has two copper ions in the active site, a mobility of one of the two copper ions was observed (Sendovski et al. 2011).

## Class II DHAP-dependent aldolases

**Scheme 2:**
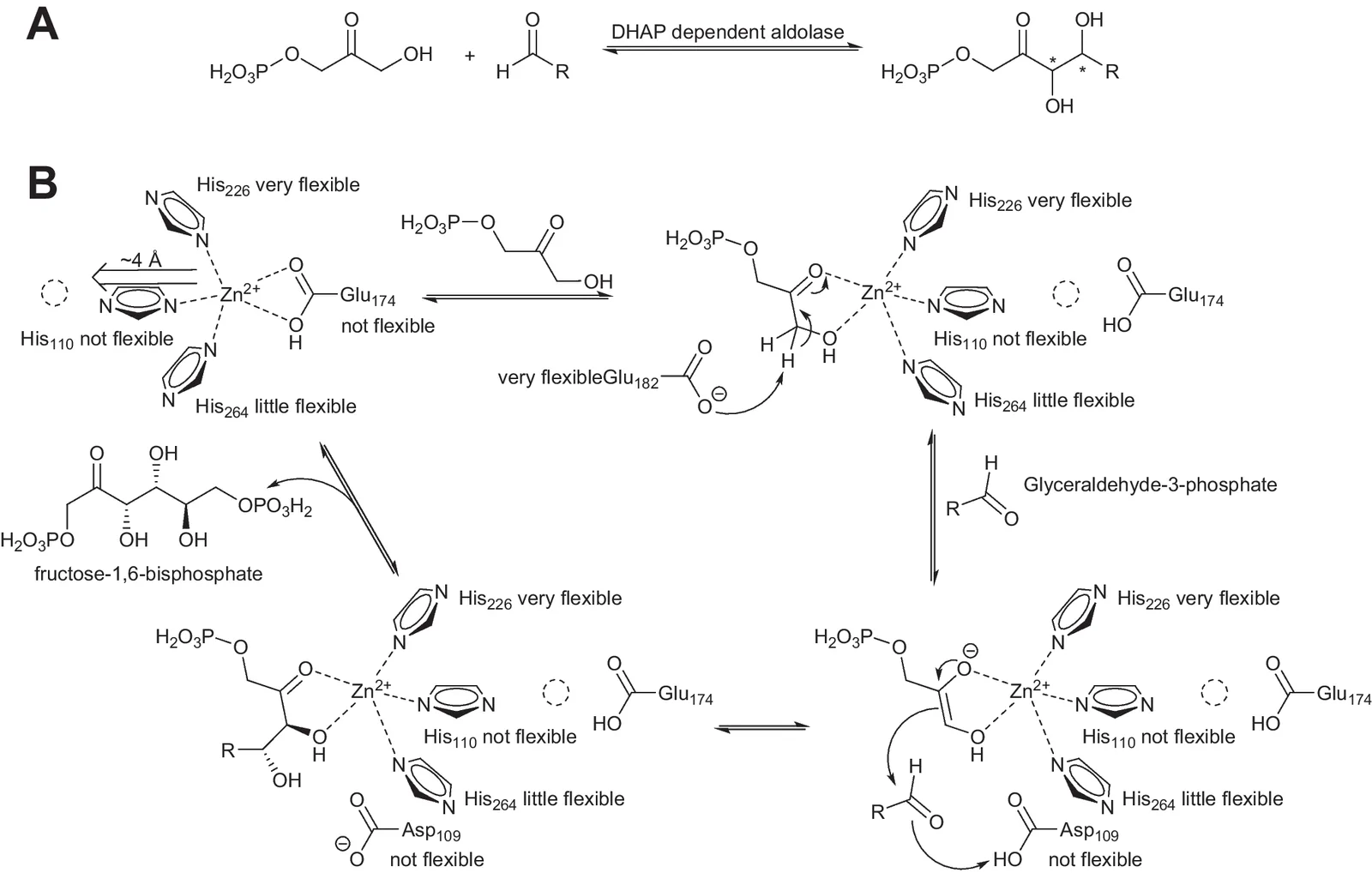
(**A**) DHAP-dependent aldolases catalyze the formation of polyhydroxyated compounds. Due to their high stereoselectivity, they can be employed for the synthesis of each of the four different stereoisomers. (**B**) Suggested reaction mechanism based on detailed studies with a range of FBPAs. The numbering given here is for the *Ec*FBPA. The remarkable flexibility of the enzyme allows the shift of the catalytic Zn(II) into its active coordination site. The Lewis acid–activated DHAP then is converted by acid base catalysis to the product FBP

Aldolases are classified into two classes according to their mechanism. Class I and class II aldolases both catalyze the same reaction, but while class I aldolases utilize an enamine mechanism, class II aldolases are metalloenzymes. The dihydroxyacetone phosphate (DHAP)–dependent aldolases play an important role in sugar metabolism and they are synthetically of great interest. In the reaction, two new stereocenters are established generating four different stereoisomers. Due to their excellent stereoselectivity, the aldolases allow the selective synthesis of each stereoisomer (Scheme 2A). The class II DHAP-dependent aldolases employ divalent metal ions as Lewis acids that bind and activate the donor molecule, here DHAP. In the vast majority of the cases, the metal is Zn(II) (Blom et al. 1996; Brovetto et al. 2011; Clapés 2015; Hanefeld et al. 2022; Hélaine et al. 2022; Ranoux 2013; Sukumaran and Hanefeld 2005).

The focus of the mechanistic studies concentrates on the class II fructose-1,6-bisphosphate aldolase (FBPA), central to sugar metabolism. This metal-dependent enzyme occurs in many pathogens, while the corresponding human aldolase is a class I enzyme with a catalytic lysine. The bacterial enzyme is consequently an ideal drug target and has been investigated in many pathogens (Capodagli et al. 2014a, b; Daher et al. 2010; Fonvielle et al. 2008; Pegan et al. 2009). In a study on the *Escherichia coli* FBPA (*Ec*FBPA), it was noted that two mutually exclusive Zn(II) binding sites were present and that the binding of Zn(II) in either site also required a significant rearrangement of the side chains with the histidine ligands. This isomerization was suggested to allow for control of the catalytic activity by switching between a buried and an exposed site (Blom et al. 1996).

Spurred by this unique structure (Blom et al. 1996), *Ec*FBPA (Hall et al. 1999; Tran et al. 2016; Zgiby et al. 2002), *Giardia lamblia* FBPA (*Gl*FBPA)(Galkin et al. 2009), and *Helicobacter pylori* FBPA (*Hp*FBPA) were studied in detail (Jacques et al. 2018). These studies revealed the putative mechanism (Scheme 2B) and considerable structural flexibility in all these FBPA’s. Due to this flexibility, a part of the crystallographic structures show a significant degree of disorder. In the buried site, the Zn(II) is coordinated by His110, 226, and 264 and by Glu174. The numbering of the residues here is that of *Ec*FBPA. The stuctural flexibility includes the shift of the catalytic Zn(II) by ~ 4 Å and a rearrangement of the coordinating histidines 226 (on a flexible loop), 264 (itself flexible), and 110 (rigid) upon substrate docking. The DHAP is a bidentate ligand of Zn(II). The metal activates the carbonyl group and decreases the pK_a_ of the proton at the alpha carbon (C-3) by a factor 10 to below 9 (Jacques et al. 2018; Kimura et al. 1999). Subsequently, Glu182 from a very flexible loop deprotonates the DHAP and carbon-carbon bond formation can take place. Nucleophilic attack of the enolate on the acceptor glyceraldehyde-3-phosphate (G3P) is enabled by concomitant protonation of the evolving hydroxy group by Asp109. The fructose-1,6-bisphosphate (FBP) then leaves the active site and the Zn(II) returns to its original buried coordination site.

**Fig. 2:**
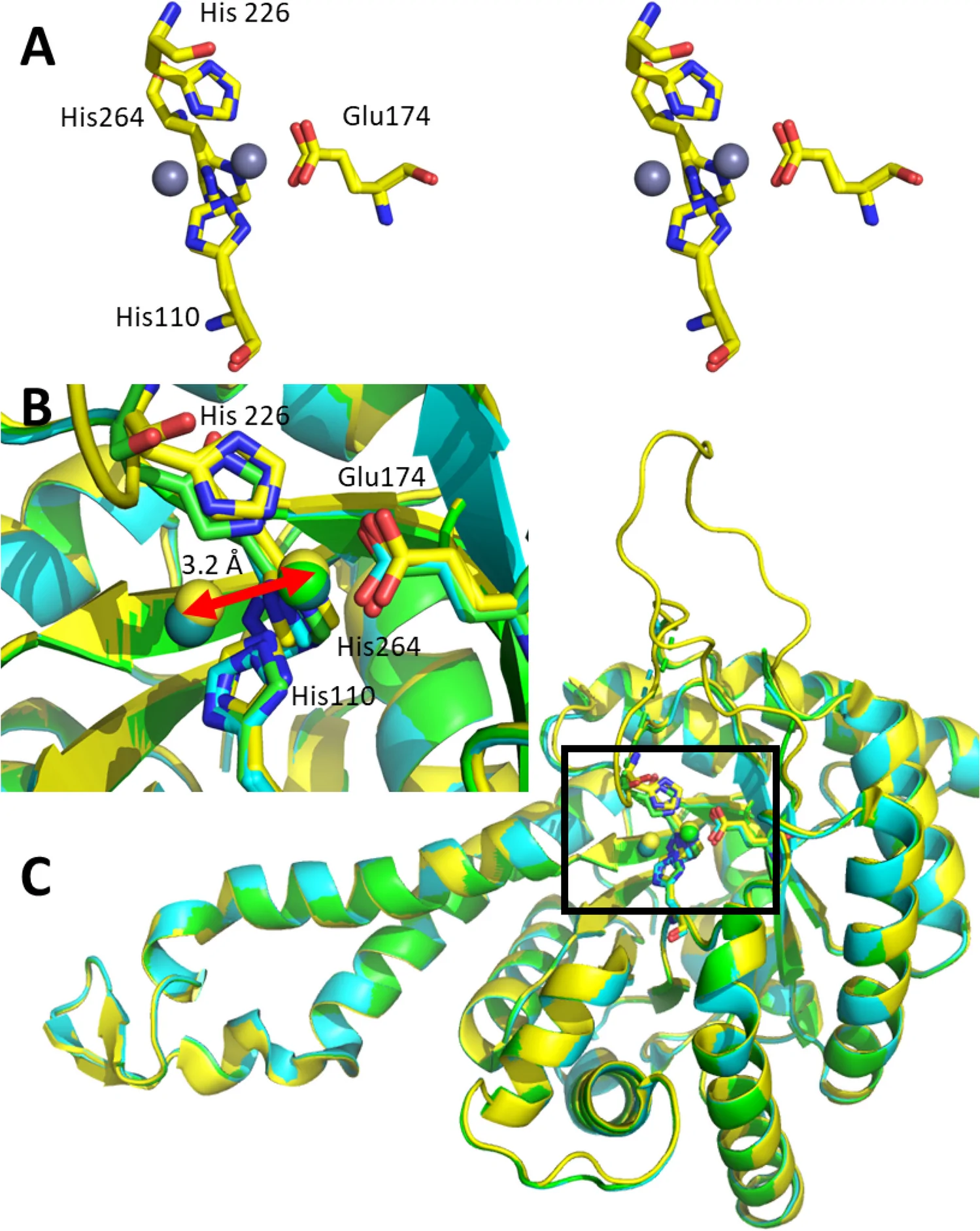
*Ec*FBPA was crystallized and the Zn(II) was found in mutually exclusive binding sites. (**A**) Active site in wall-eyed stereoview of PDB-ID 1DOS (yellow, Zn grey) with a metal shift of 3.2 Å. The imidazole ring of His110 is flipped, as is the imidazole ring of His226; His264 is located in two different position, however less extreme than the Zn(II). (**B**) Active site of *Ec*FBPA, overlay of PDB-ID 1DOS (yellow), 5GK3 (green), and 5GK4 (blue). In 1DOS and 5GK4, the Zn(II) is distributed of both mutually exclusive binding sites. Correspondingly, the imidazole ring of His110 is flipped and His264 is located in two different position. Due to the high flexibility of the side loop, His226 is not visible in 5GK4. In 5GK3, Zn(II) is only in the buried binding site and all His ligands are well-defined in a single position. (**C**) Full view of overlay of PDB-ID 1DOS, 5GK3, and 5GK4. The high flexibility of the loops Cys176 to Leu195 and Gly223 to Val234 causes them to be ill-defined

In crystallographic studies of *Ec*FBPA, the Zn(II) was in several cases not only located in the buried site with Zn coordinated by His110, 226, and 264 and by Glu174 but also exposed site coordinated only by His110, 226, and 264 (Fig. 2) (Blom et al. 1996; Tran et al. 2016). Indeed this was the case in the initial report, and can also be seen for other FBPAs. The structural flexibility is clear from the different orientations of His264 for which part of the population coordinated Zn(II) in the buried and the other in the open coordination (Fig. 2A and B). In the overlay, it is evident that in particular the loops Cys176 to Leu195 and Gly223 to Val234 are ill-defined. These loops contain the residues which require high motility (Fig. 2C). As a consequence thereof, His226 is not always defined in the structures. For *Gl*FBPA, similar observations were made (Galkin et al. 2009). These structural investigations were extended to a DHAP-dependent aldolase that catalyzes the formation of octuluronate-1-phosphate from DHAP and l-arabinuronate. Again, two mutually exclusive Zn(II) binding sites were identified, one buried and one exposed (Huddleston et al. 2019).

**Fig. 3:**
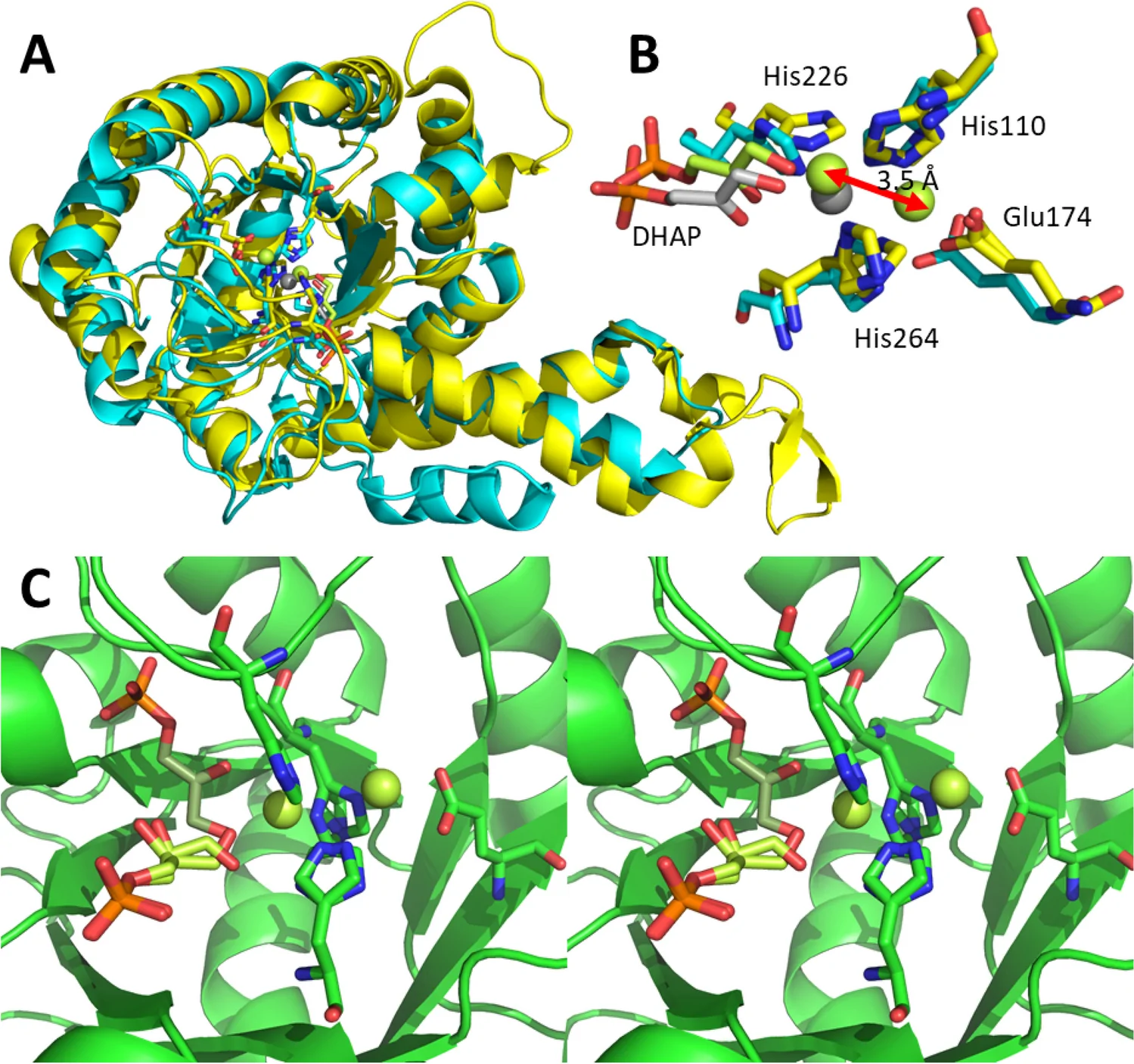
FBPA after DHAP and FBP soaking. (**A**) Overlay of *Ec*FBPA PDB-ID 5VJD (yellow, DHAP lime and Zn lime) and *Hp*FBPA PDB-ID 5VJF (blue, Zn grey and DHAP grey) both soaked with DHAP. The high flexibility of the loops Cys176 to Leu195 (in *Ec*FBPA numbering) causes them to be ill-defined. (**B**) Enlarged active site of *Ec*FBPA (PDB-ID 5VJD) and *Hp*FBPA (PDB-ID 5VJF) both soaked with DHAP. (**C**) Soaking of *Hp*FBPA with FBP led to a retro-aldol reaction in the active site. Active site in wall-eyed stereoview PDB-ID 5UCK (green, DHAP smudge, Zn limon and G3P limon)

In a detailed study of *Hp*FBPA, it was demonstrated that the same residues were essential for catalysis. In direct comparison to *Ec*FBPA, both crystals were soaked with DHAP (Jacques et al. 2018). The same loop (Cys176 to Leu195 in *Ec*FBPA numbering) was disordered. DHAP in both cases coordinated as bidentate ligand to the Zn(II) in the open coordination site (Fig. 3A and B). While in *Ec*FBPA also here displays partial Zn(II) occupation of the buried site, *Hp*FBPA has all the metal in the active position (Fig. 3A and B). When soaking *Hp*FBAP with the product FBP, the retro-aldol reaction took place in the crystal (Fig. 3C). The enediolate from DHAP is oriented such that the nucleophilic attack will take place from its *si* face onto the *si*-face of G3P. A small fraction of the crystal is populated by a G3P with reversed aldehyde group, i.e. the *re*-face is oriented towards the DHAP binding site. In this case, the Zn(II) is in the buried binding site.

**Scheme 3:**
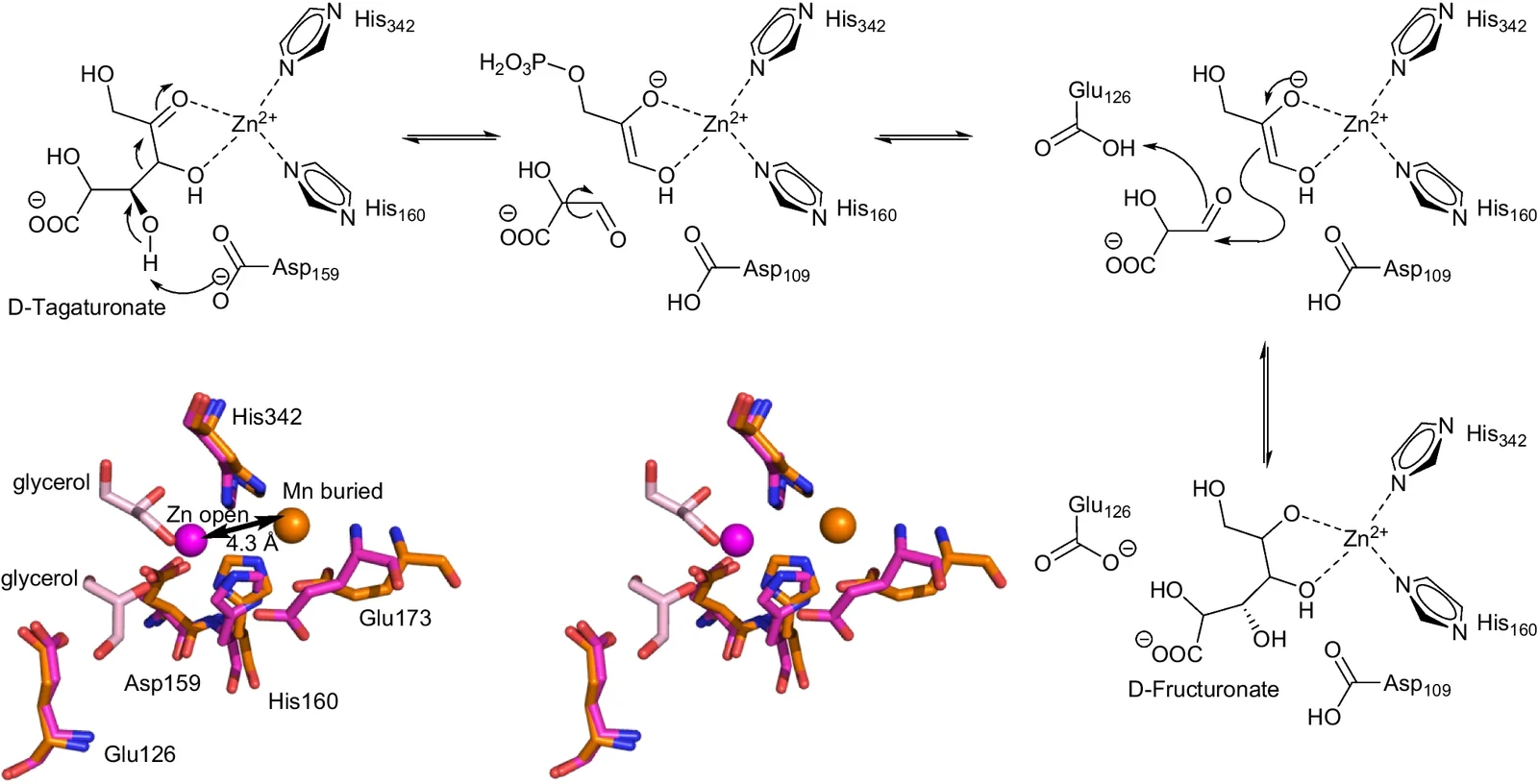
Proposed reaction mechanism for the epimerase UxaE from *Cohnella laeviribosi*. The retro-aldol reaction, subsequent rotation of the aldehyde group, and aldol reaction epimerize the hydroxy group at C-3. The stereochemistry is given only for the epimerized hydroxy group. Inset: Active site in wall-eyed stereoview. Overlay with the metal, Mn(II), in the buried site (PDB-ID 6ILB, orange) and Zn(II) in the open site (PDP IB 6ILA, magenta with the glycerol molecules in light pink)

The fact that G3P can dock in two different orientations is of interest, as it would explain the occasionally observed lower stereospecificity of aldolases for this carbon (Marsden et al. 2020, 2021). This relaxed stereospecificity at the same time seems to be the functional basis of epimerase UxaE from *Cohnella laeviribosi* (Choi et al. 2020). This enzyme utilizes Zn(II) and similar divalent metals such as Mn(II) as cofactor and the proposed mechanism makes use of the relaxed docking of the aldehyde, such as in *Hp*FBAP. d-Tagaturonate is deprotonated by Asp159 and a retro-aldol reaction occurs. The intermediate aldehyde group rotates and is now nucleophilically attacked by the enediolate. Glu126 protonates the evolving alcoholate and C-3 of d-tagaturonate is thus epimerized to d-fructuronate (Scheme 3). The active metal is in the exposed coordination during this process but otherwise is in the buried position. The buried position is clearly visible in a structure with Mn(II); no substrates are coordinated. When Zn(II) was used and glycerol was added as a mimic for dihydroxyacetone, the Zn(II) shifted by up to 4.3 Å to the open coordination site (Scheme 3).

## Zn^2+^-dependent medium-chain dehydrogenase/reductase ADH

**Fig. 4:**
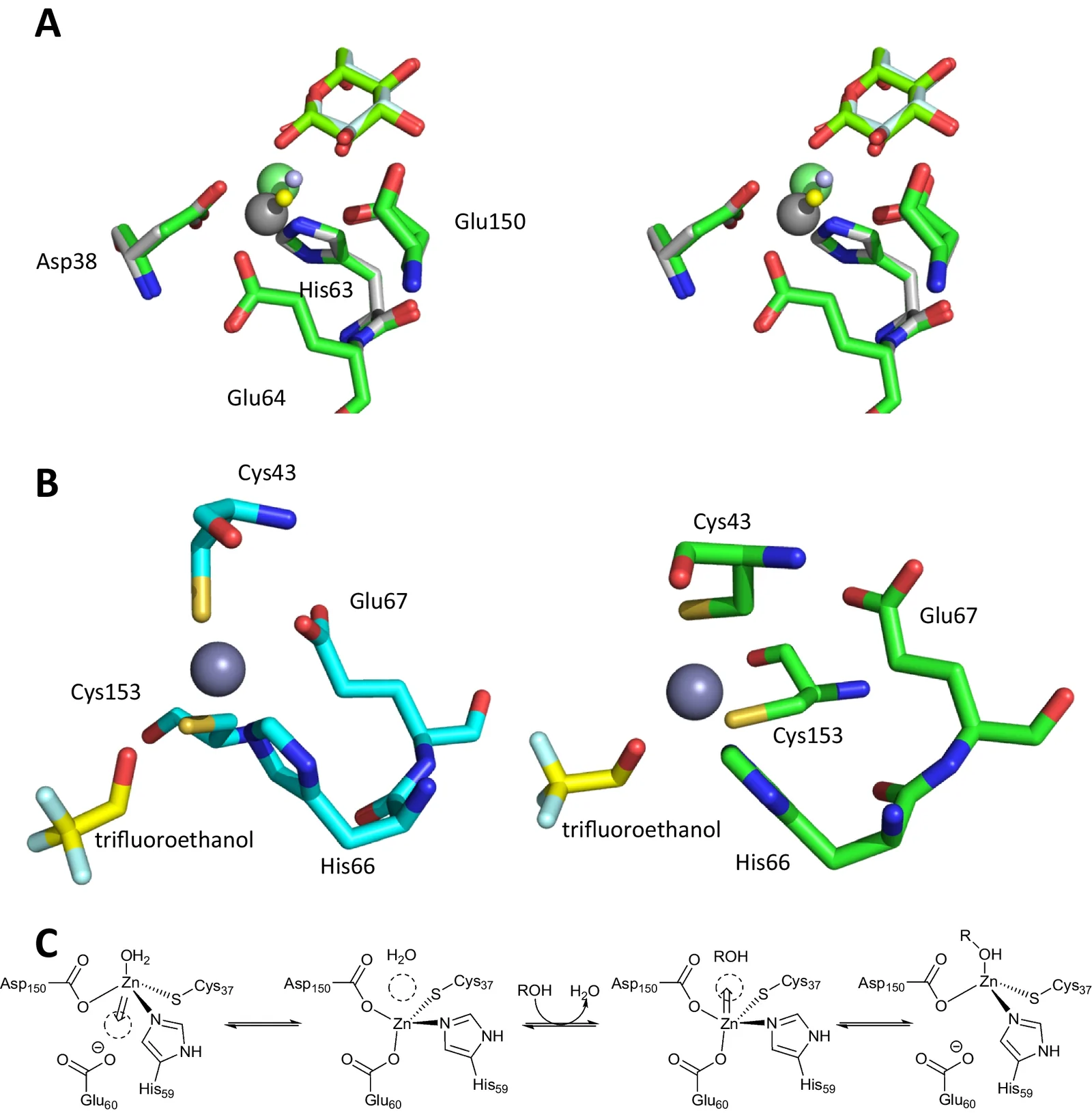
(**A**) The *Hm*GDH displays a remarkable movement of Zn(II) and the water coordinated to it, depending on the oxidation state of the substrate. Active site in wall-eyed stereoview of the overlay of enzyme with product PDB-ID 2VWG, grey, gluconolactone palecyan, Zn(II) grey and water light blue and with starting material PDB-ID 2VWH green, glucose chartreuse, Zn green and water yellow. The coordinating residues virtually do not move, due to this Glu64 and Glu150 (two alternative conformations) of 2VWG are essentially not visible. (**B**) For *Sc*ADH1, the two subunits show different coordination of the Zn(II) in the presence of the substrate trifluoroethanol (yellow in both structures). In the structure PDB-ID 5ENV in subunit B (on the left side in blue with Zn(II) tetrahedrally coordinated by Cys43, His66, Glu67, and Cys153, the metal is coordinated only by the enzyme. On the right side in green subunit A, the trifluoroethanol replaced Glu67 in the tetrahedral coordination of Zn(II). All ligands and the Zn(II) have moved relative to subunit B. (**C**) The double inversion of the Zn(II) leads to the coordination of the alcohol in MDR’s. The numbering of the residues is in accordance with *Tp*ADH, the protonation states of Cys37 and Asp150 are not yet defined

The medium-chain dehydrogenases/reductases (MDR) family typically has a Zn(II) in tetrahedral coordination. This Zn(II) is coordinated by a variety of amino acid residues, the imidazole of histidine, the thiol of cysteine, and the acid groups of aspartic or glutamic acid being the most common. Often one site is occupied by water and this has been speculated to occur as water or hydroxide ion. It was noted that in the course of the reaction, the Zn(II) shifts position (Sanghani et al. 2002). This shift of 1–2.3 Å is essential to enable catalysis and is linked both to improved Lewis acidity for the dehydrogenation step and increased alkalinity for the hydride attack (Baker et al. 2009; Guntupalli et al. 2021; Plapp et al. 2017; Sellés Vidal et al. 2018). In the human glutathione-dependent formaldehyde dehydrogenase (FDH), the shift was associated also with the conformational changes due to the nicotinamide cofactor entering and leaving the active site (Sanghani et al. 2002). In the glucose dehydrogenase from *Haloferax mediterranei* (*Hm*GDH), these structural changes in the Zn(II) coordination were reported to occur while the protein ligands did not move. As part of the protein relay, it is proposed that a hydroxide is coordinated to the Zn(II) that can take up the proton of the hydroxy group of the substrate when this is oxidized. Both the Zn(II) and the water/hydroxide change position (Fig. 4A). The movement of Zn(II) here is approx. 1.3 Å.

The mechanistic role of Zn(II) has been studied in detail in other ADH enzymes of the MDR family, such as *Saccharomyces cerevisiae* ADH1 (*Sc*ADH1) (Guntupalli et al. 2021; Plapp et al. 2016), horse liver ADH1E (HlADH) (Plapp et al. 2017), and *Thermoanaerobacter pseudoethanolicus* ADH (*Tp*ADH)(Dinh et al. 2022). In *Sc*ADH1, the Zn(II) is tetrahedrally coordinated and the two structures observed in the subunits indicate a double inversion of the Zn(II) during coordination; first, the coordinated water is released and replaced by glutamate; subsequently, the second inversion leads to the coordination of the substrate alcohol in the position previously taken by water (Fig. 4B). This occurs with a concomitant movement of Zn(II) and of the coordinating amino acid residues. The examination of HlADH includes a careful discussion of MDRs in general for each reaction step and concludes that a double displacement mechanism via a transition state with trigonal bipyramidal geometry is likely (Fig. 4C). At the same time, it is concluded that a penta-coordinated intermediate is unlikely. Interestingly, the double displacement mechanism is supported by the investigation of *Tp*ADH. When soaking the crystals of *Tp*ADH, tetrahedral complexes with the alcohol as a ligand were observed, similar to the coordination of trifluoroethanol in the active site of *Sc*ADH1 (Fig. 4B right). For *Tp*ADH, distorted penta-coordinated Zn(II) was also found when the crystals were soaked with (1*S*,3*S*)-3-methylcyclohexanol. This would support a trigonal bipyramidal transition state.

Overall, the picture arises that the MDR are rather flexible to allow NAD^+^/NADH exchange. The coordination of the substrate takes place via a double displacement, i.e. two S_N_2 reaction at the Zn(II) with rearrangement of the Zn(II) and the coordinating amino acid residues (Fig. 4B and C). In most cases, the Zn(II) has three amino acid residues and the substrate coordinated to it when the actual reaction occurs and the amino acids are rigid. However, if Zn(II) is coordinated by two amino acid residues, the substrate and water/hydroxide, then a shift of the metal and the water/hydroxide occurs during the reaction (Fig. 4A).

## Class II pyruvate-dependent aldolases

**Fig. 5:**
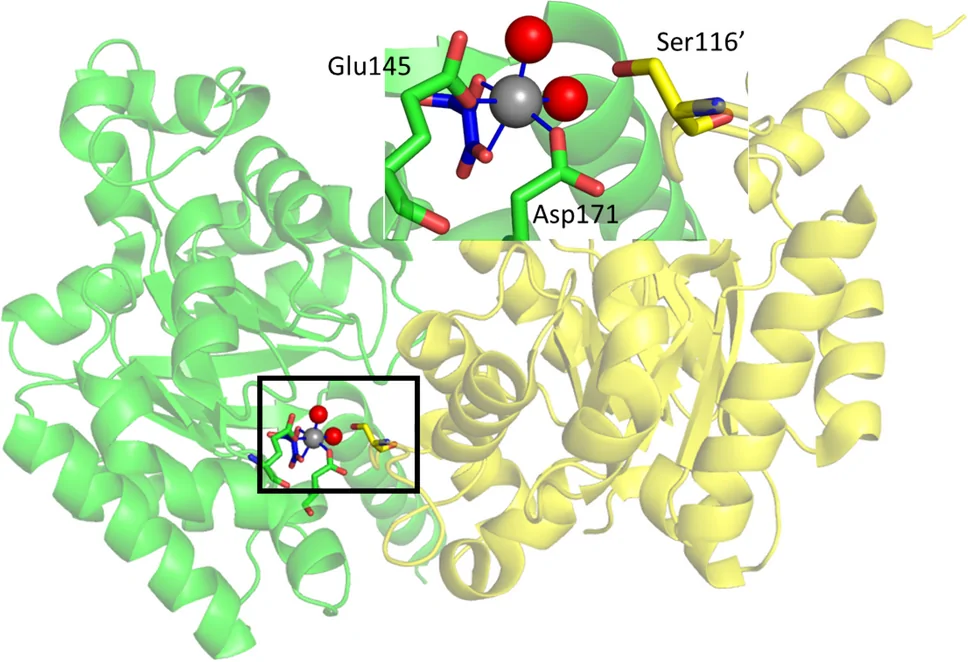
The *Sw*HKA crystal was soaked with HPA (PDB-ID 6R62). Two subunits, green and yellow, of the hexameric *Sw*HKA with the active site located at their interface are shown. HPA (blue) coordinates to Mg(II) (grey) as do two acidic residues of the green subunit. The serine of the adjacent subunit is not involved in metal coordination. Inset: enlargement of the active site

As DHAP-dependent aldolases (see above), pyruvate-dependent aldolases (PDA) utilize two different mechanisms and are classified by them as class I and II. The class II PDA typically contain divalent metal ions, ranging from Mg(II) to Co(II). They have recently been studied for their synthetic utility with a focus on expanding the range of donor molecules beyond pyruvate (de Berardinis et al. 2017; Fang et al. 2019; Gastaldi et al. 2021; Laurent et al. 2020; Marsden et al. 2019). When studying *Sphingomonas wittichii* RW1 hydroketoacid aldoxy lase (*Sw*HKA; recently the name of the organism was altered to *Rhizorhabdus wittichii* RW1), the active site was found to be located at the interface of two subunits, with six active sites in the trimer of the homodimers (Fig. 5). The hydroxy pyruvic acid (HPA) coordinates as bidentate ligand to the Mg(II), and the other four positions are taken by two acidic residues, Glu145 and Asp171 and two water molecules (Marsden et al. 2019, 2022). Ser116’ from the other subunit is close by and completes the active site. This coordination is not uniform for PDA (Coincon et al. 2012).

Careful comparison of the enzyme with and without substrate soaking revealed a resting state of the enzyme with the catalytic metal Mn(II) or Mg(II) coordinated 2.3 Å away from the active position. The hexacoordinated metal is now also attached to the Ser116’ of the neighbouring subunit. Again the two acidic residues, Glu145 and Asp171, are coordinated to the metal, as are three water molecules. Mutational and stability studies revealed that the metal does not contribute to the stabilization of the dimer (Marsden et al. 2022).

**Fig. 6:**
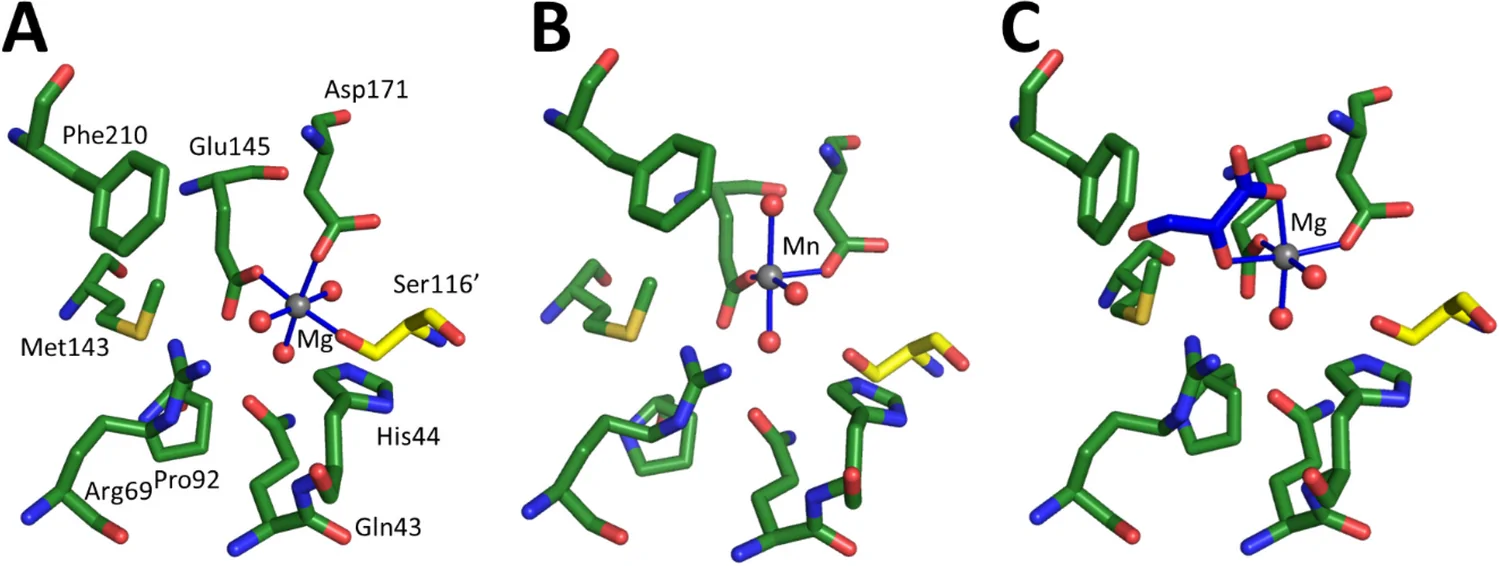
Resting and active state coordination in *Sw*HKA. (**A**) Octahedral M(II) coordination of the metal cofactor at the interface between two dimers (PDB-ID 7NUJ dark green and yellow for the subunits, the metal is Mg(II)). This structure with M(II) was observed in the following crystals structures: Holo-*Sw*HKA-Mg(II), Holo-*Sw*HKA-Mn(II), Holo-F210W-Mg(II), Holo-H44A-Mg(II) - PDB-ID 7NUJ; 7O5R; 7O5W; 7O9R, respectively. (**B**) Square pyramidal M(II) coordination with a vacant coordination site. This complex was only observed with low occupancy of Mn(II) in the absence of substrates in the crystal structure *Sw*HKA-Mn(II) (PDB-ID 7O5R, colours as for A, the metal is Mn(II)). (C) Coordination of the catalytically active state M(II). (PDB-ID 6R62, colours and metal as for A, HPA blue). This complex was observed in the presence of HPA in *Sw*HKA-HPA-Mg(II), *Sw*HKA-F201W-HPA-Mg(II), and *Sw*HKA-S116A-HPA-Mg(II) (PDB-ID 6R62, 7O87, and 7NNK, respectively)

With Mg(II) as cofactor, the octahedral metal resided in the resting state, coordinated by three residues (Glu145, Asp171, and Ser116’) and three water molecules (Fig. 6A). The addition of the substrate HPA causes a shift to the active coordination site (Fig. 6C). Now, the octahedral Mg(II) is coordinated by Glu145, Asp171, HPA, and two water molecules. For Mn(II), a low occupancy of an intermediate position with a penta-coordinated metal was observed (Fig. 6B). Also here, only two amino acid residues, Glu145 and Asp171, bind the metal to the enzyme while the other three places are taken up by water molecules. This shift between resting and active position of the metal in the active site is similar to the movement of Zn(II) in the DHAP-dependent aldolases. However, the protein scaffold of the HKA is static.

## The distribution of metal mobility among enzyme families and taxonomies

The above examples convincingly demonstrate the use of metal mobility and their underlying mechanisms in various metalloenzymes. However, only a few examples have been studied in detail from each enzyme family. In order to investigate whether metal mobility in catalytic conversions is a more general feature or rather an exception from metals that are stationary throughout the catalytic cycle, we performed a structure motif search using the crystal structures of the aforementioned enzymes and the RCSB PDB (Bittrich et al. 2020).

For d-xylose isomerase, P15587 (PDB-ID 1XYA) from *Streptomyces olivochromogenes* and the residues Glu216, His219, Asp254, Asp256 including Glu180 (which is from the second metal binding site) were selected. Secondly, for fructose-bisphosphate aldolase class II, the protein P0AB71 (PDB-ID 5GK3) from *Escherichia coli* K12 was selected, and the residues His110, His226, His264, and Glu174. Finally, for Zn(II)-dependent medium-chain dehydrogenase/reductase ADH, B0KBL1 (PDB-ID 7UX4) from *Thermoanaerobacter pseudethanolicus* was selected, including the residues Cys37, His59, Glu60, and Asp150. For all searches, Asp and Glu were set to be interchangeable, while default settings were used otherwise. Unfortunately, for the class II pyruvate aldolase such as the HKA from *Rhizorhabdus wittichii* RW1 (formerly *Sphingomonas wittichii*; *Sw*HKA), the active site is located at the interface of two subunits, which did not allow for a comparable search to be performed as with the other enzyme templates.

**Fig. 7:**
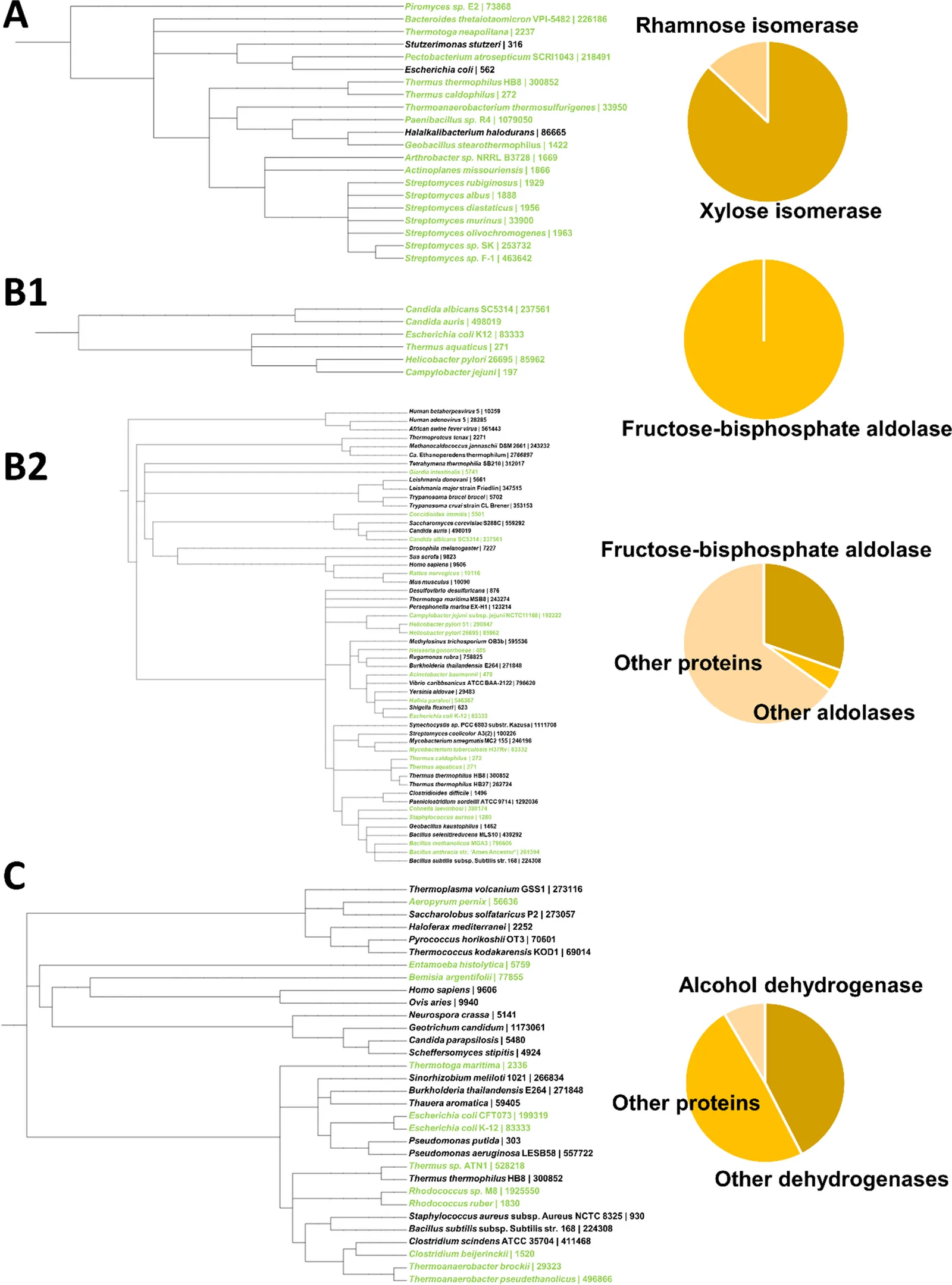
Phylogenetic distribution of structure motif search matches in the RCSB PDB using the crystal structures (PDB-ID 1XYA, 5GK3, and 7UX4) of the enzymes discussed above

Interestingly, the search for XI revealed several additional XIs across the *Terrabacteria* group, including one from a fungus, but also a few Rhamnose isomerases from the genera *Escherichia*, *Stutzerimonas*, and *Halalkalibacterium* (Fig. 7A). Although this binding motif appears to be present across different bacterial phyla, it seems to be conserved in xylose and a few rhamnose isomerases.

The search with the FBPA class II motif template returned two FBPAs from the genus *Candida*, and a few more bacterial species (Fig. 7B). When excluding the very flexible branch with the His226 residue from the search, many more entries were returned, including other sugar bisphosphate aldolases. However, also a range of unrelated RNA and DNA binding proteins indicate that the search parameters were likely already too unspecific. Nevertheless, this binding motif is present in several different species, but seems to be more prevalent in FBPAs.

Finally, for the ADH from *Thermoanaerobacter pseudoethanolicus*, matches were obtained from across all domains of life (Fig. 7C). Interestingly, in addition to several different alcohol dehydrogenases, also matches with sorbitol dehydrogenase (*Homo sapiens*), glucose dehydrogenase (*Thermoplasma volcanium* GSS1), arabinose dehydrogenase (*Saccharolobus solfataricus* P2), and formaldehyde dehydrogenase (*Pseudomonas putida*) were identified. This suggests that the binding centre found in the ADH of *Thermoanaerobacter pseudoethanolicus* is present across a spectrum of dehydrogenases, throughout the tree of life. This is in line with the different MDR family enzymes described under the corresponding section.

## Conclusions

To date, the vast majority of all metalloenzyme are considered to have a static metal cofactor that does not move prior or during catalysis. Exploring the field a number of different enzymes is found to display metal cofactor movement. In all the cases of metal movement known, the metal cofactor is directly complexed by the protein. The variety in the metal movement is surprising, clearly nature has found solutions suiting each single case.

In the XIs and the MDRs, the metal movement clearly is essential during the catalytic cycle. The reaction requires the movement of the Mg-2 in XI to enable the hydride shift catalysis to take place. This occurs while the protein remains almost stationary. In MDRs, the movement of the Lewis acid is also essential to dock the substrate and even during the catalytic cycle. Concerning the protein, it displays different behaviour to XI, as both the metal and the protein scaffold move for the double inversion at the metal cofactor of MDRs to take place. In view of the conformational changes linked to the NAD(P)H binding, this is not surprising. In both cases, the plasticity of the metal cofactor enables catalysis and thus provides an evolutionary advantage.

For the class II aldolases, a different reason for metal movement is observed. The cofactor is either in a resting or buried state, complexed by more amino acid residues. Upon docking of the substrate, it moves to more open active state. Here, the substrates DHAP and pyruvate act as bidentate ligands and the metal cofactor is less tightly coordinated to the protein. In the DHAP-dependent aldolases, metal shift occurs with a rearrangement of the coordinating amino acid residues and the scaffold displays great flexibility. In the PDA, the enzyme scaffold is static. However, in both cases, the shift from a more coordinated resting state to a less coordinated active state indicates that the metal shift might have evolved as a trade-off between optimal metal cofactor stability and catalytic plasticity.

The mobility of the metal cofactor can lead to evolutionary advantages was demonstrated for paraoxonase-1 (PON1). The wild-type enzyme catalyzes the hydrolysis of lipophilic lactones. The Ca(II) cofactor can however shift 1.8 Å and then enables a promiscuous reaction: the breakdown of organophosphates like paraoxon. Mutational studies were conducted to further developed this promiscuous activity (Ben-David et al. 2013).

Structural motif searches show that the examples reported here are not rare cases, but may be far more prevalent in nature. Overall, it can be expected that the movement of the metal cofactor in metalloenzymes plays a more important role than perceived up to date.
